# Some Evidence on the Cognitive Benefits of Learning to Code

**DOI:** 10.3389/fpsyg.2021.559424

**Published:** 2021-09-09

**Authors:** Ronny Scherer, Fazilat Siddiq, Bárbara Sánchez-Scherer

**Affiliations:** ^1^Centre for Educational Measurement, Faculty of Educational Sciences, University of Oslo, Oslo, Norway; ^2^Department of Education and Quality in Learning, Unit for Digitalisation and Education, Kongsberg, Norway; ^3^Department of Biology, Humboldt University of Berlin, Berlin, Germany

**Keywords:** computational thinking skills, transfer of learning, cognitive skills, meta-analysis, experimental studies

## Introduction

Computer coding—an activity that involves the creation, modification, and implementation of computer code and exposes students to computational thinking—is an integral part of today's education in science, technology, engineering, and mathematics (STEM) (Grover and Pea, 2013). As technology is advancing, coding is becoming a necessary process and much-needed skill to solve complex scientific problems efficiently and reproducibly, ultimately elevating the careers of those who master the skill. With many countries around the world launching coding initiatives and integrating computational thinking into the curricula of higher education, secondary education, primary education, and kindergarten, the question arises, what lies behind this enthusiasm for learning to code? Part of the reasoning is that learning to code may ultimately aid students' learning and acquiring of skills in domains other than coding. Researchers, policy-makers, and leaders in the field of computer science and education have made ample use of this argument to attract students into computer science, bring to attention the need for skilled programmers, and make coding compulsory for students. Bill Gates once stated that “[l]earning to write programs stretches your mind, and helps you think better, creates a way of thinking about things that I think is helpful in all domains” (2013). Similar to the claims surrounding chess instruction, learning Latin, video gaming, and brain training (Sala and Gobet, 2017), this so-called “transfer effect” assumes that students learn a set of skills during coding instruction that are also relevant for solving problems in mathematics, science, and other contexts. Despite this assumption and the claims surrounding transfer effects, the evidence backing them seems to stand on shaky legs—a recently published paper even claimed that such evidence does not exist at all (Denning, 2017), yet without reviewing the extant body of empirical studies on the matter. Moreover, simply teaching coding does not ensure that students are able to transfer the knowledge and skills they have gained to other situations and contexts—in fact, instruction needs to be designed for fostering this transfer (Grover and Pea, 2018).

In this opinion paper, we (a) argue that learning to code involves thinking processes similar to those in other domains, such as mathematical modeling and creative problem solving, (b) highlight the empirical evidence on the cognitive benefits of learning computer coding that has bearing on this long-standing debate, and (c) describe several criteria for documenting these benefits (i.e., transfer effects). Despite the positive evidence suggesting that these benefits may exist, we argue that the transfer debate has not yet to be settled.

## Computer Coding as Problem Solving

Computer coding comprises activities to create, modify, and evaluate computer code along with the knowledge about coding concepts and procedures (Tondeur et al., 2019). Ultimately, computer science educators consider it a vehicle to teaching computational thinking through, for instance, (a) abstraction and pattern generalization, (b) systematic information processing, (c) symbol systems and representations, (d) algorithmic thinking, (e) problem decomposition, (f) debugging and systematic error detection (Grover and Pea, 2013). These skills share considerable similarities with general problem solving and problem solving in specific domains (Shute et al., 2017). Drawing from the “theory of common elements,” one may therefore expect possible transfer effects between coding and problem solving skills (Thorndike and Woodworth, 1901). For instance, creative problem solving requires students to encode, recognize, and formulate the problem (preparation phase), represent the problem (incubation phase), search for and find solutions (illumination phase), evaluate the creative product and monitor the process of creative activities (verification phase)—activities that also play a critical role in coding (Clements, 1995; Grover and Pea, 2013). Similarly, solving problems through mathematical modeling requires students to decompose a problem into its parts (e.g., variables), understand their relations (e.g., functions), use mathematical symbols to represent these relations (e.g., equations), and apply algorithms to obtain a solution—activities mimicking the coding process. These two examples illustrate that the processes involved in coding are close to those involved in performing skills outside the coding domain (Popat and Starkey, 2019). This observation has motivated researchers and educators to hypothesize transfer effects of learning to code, and, in fact, some studies found positive correlations between coding skills and other skills, such as information processing, reasoning, and mathematical skills (Shute et al., 2017). Nevertheless, despite the conceptual backing of such transfer effects, which evidence exists to back them empirically?

## Cognitive Benefits of Learning Computer Coding

Despite the conceptual argument that computer coding engages students in general problem-solving activities and may ultimately be beneficial for acquiring cognitive skills beyond coding, the empirical evidence backing these transfer effects is diverse (Denning, 2017). While some experimental and quasi-experimental studies documented mediocre to large effects of coding interventions on skills such as reasoning, creative thinking, and mathematical modeling, other studies did not find support for any transfer effect. Several research syntheses were therefore aimed at clarifying and explaining this diversity.

In 1991, Liao and Bright reviewed 65 empirical studies on the effects of learning-to-code interventions on measures of cognitive skills (Liao and Bright, 1991). Drawing from the published literature between 1960 and 1989, the authors included experimental, quasi-experimental, and pre-experimental studies in classrooms with a control group (non-programming) and a treatment group (programming). The primary studies had to provide quantitative information about the effectiveness of the interventions on a broad range of cognitive skills, such as planning, reasoning, and metacognition. Studies that presented only correlations between programming and other cognitive skills were excluded. The interventions focused on learning the programming languages Logo, BASIC, Pascal, and mixtures thereof. This meta-analysis resulted in a positive effect size quantified as the well-known Cohen's *d* coefficient, indicating that control group and experimental group average gains in cognitive skills differed by 0.41 standard deviations. Supporting the existence of transfer effects, this evidence indicated that learning coding aided the acquisition of other skills to a considerable extent. Although this meta-analysis was ground-breaking at the time, transferring it into today's perspective on coding and transfer is problematic for several reasons: First, during the last three decades, the tools used to engage students in computer coding have changed substantially, and visual programming languages such as Scratch simplify the creation and understanding of computer code. Second, Liao and Bright included any cognitive outcome variable without considering possible differences in the transfer effects between skills (e.g., reasoning may be enhanced more than reading skills). Acknowledging this limitation, Liao (2000) performed a second, updated meta-analysis in 2000 summarizing the results of 22 studies and found strong effects on coding skills ($\bar{d}$ = 2.48), yet insignificant effects on creative thinking ($\bar{d}$ = −0.13). Moderate effects occurred for critical thinking, reasoning, and spatial skills ($\bar{d}$ = 0.37–0.58).

**Figure 1:**
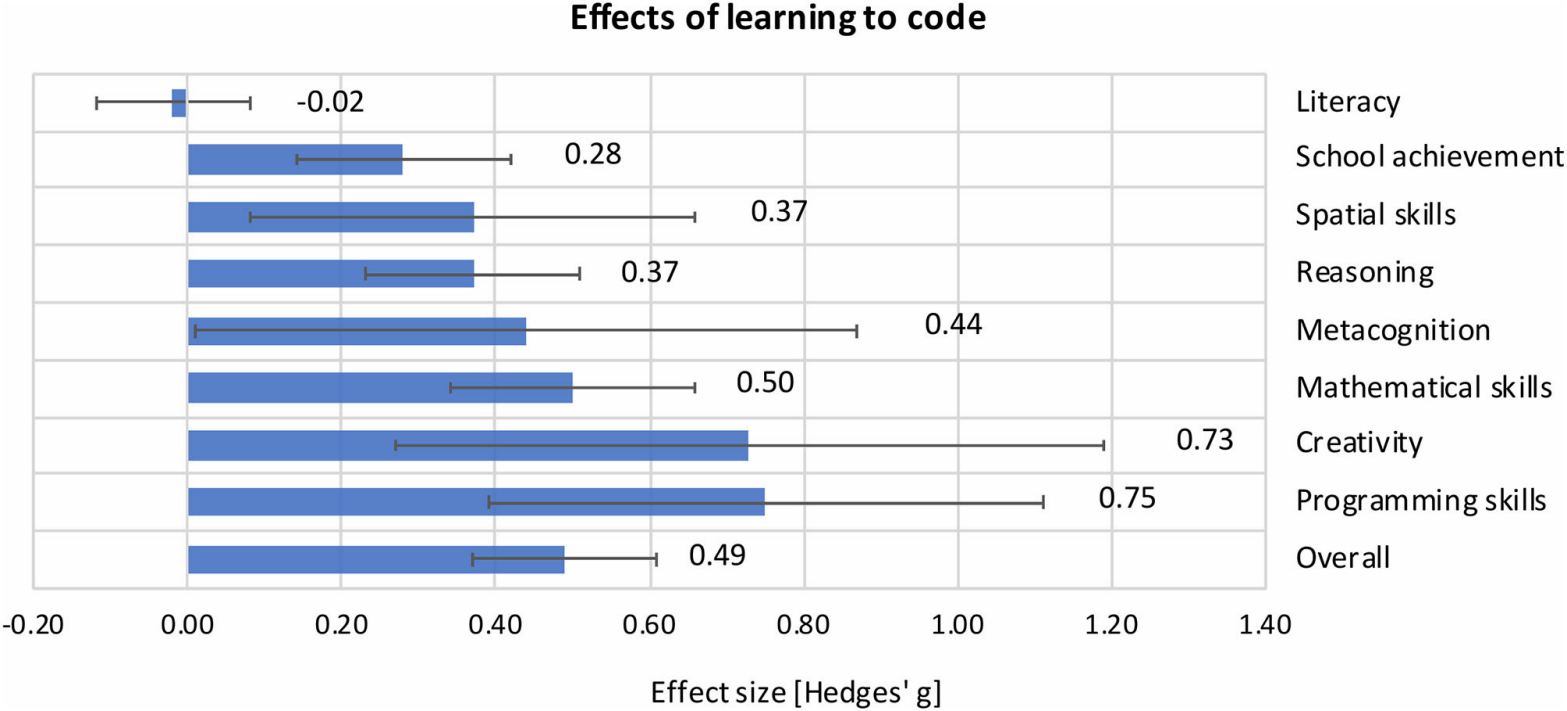
Effect sizes of learning-to-code interventions on several cognitive skills and their 95% confidence intervals (Tondeur et al., 2019). The effect sizes represent mean differences in the cognitive skill gains between the control and experimental groups in units of standard deviations (Hedges' *g*).

Drawing from a pool of 105 intervention studies and 539 reported effects, Tondeur et al. (2019) put the question of transfer effects to a new test. Their meta-analysis included experimental and quasi-experimental intervention studies with pretest-posttest and posttest-only designs. Each educational intervention had to include at least one control group and at least one treatment group with a design that allowed for studying the effects of coding (e.g., treatment group: intervention program of coding with Scratch®, control group: no coding intervention at all; please see the meta-analysis for more examples of study designs). Finally, the outcome measures were performance-based measures, such as the Torrance Test of Creative Thinking or intelligence tests. This meta-analysis showed that learning to code had a positive and strong effect on coding skills ($\bar{g}$ = 0.75) and a positive and medium effect on cognitive skills other than coding ($\bar{g}$ = 0.47). The authors distinguished further between the different types of cognitive skills and found a range of effect sizes, $\bar{g}$ = −0.02–0.73 (Figure 1). Ultimately, they documented the largest effects for creative thinking, mathematical skills, metacognition, reasoning, and spatial skills ($\bar{g}$ = 0.37–0.73). At the same time, these effects were context-specific and depended on the study design features, such as randomization and the treatment of control groups.

These research syntheses provide some evidence for the transfer effects of learning to code on other cognitive skills—learning to code may indeed have cognitive benefits. At the same time, as the evidence base included some study designs that deviated from randomized controlled trials, strictly causal conclusions (e.g., “Students' gains in creativity were caused by the coding intervention.”) cannot be drawn. Instead, one may conclude that learning to code *was associated* with improvements in other skills measures. Moreover, the evidence does not indicate that transfer just “happens”; yet, it must be facilitated and trained explicitly (Grover and Pea, 2018). This represents a “cost” of transfer in the context of coding: among others, teaching for transfer requires sufficient teaching time, student-centered, cognitively activating, supportive, and motivating learning environments, and teacher training—in fact, possible transfer effects can be moderated by these instructional conditions (e.g., Gegenfurtner, 2011; Yadav et al., 2017; Waite et al., 2020; Beege et al., 2021). The extant body of research on fostering computational thinking through teaching programming suggests that problem-based learning approaches that involve information processing, scaffolding, and reflection activities are effective ways to promote the near transfer of coding (Lye and Koh, 2014; Hsu et al., 2018). Beside the cost of effective instructional designs, another cost refers to the cognitive demands of the transfer: existing models of transfer suggest that the more similar the tasks during the instruction in one domain (e.g., coding) are to those in another domain (e.g., mathematical problem solving), the more likely students can transfer their knowledge and skills between domains (Taatgen, 2013). Mastering this transfer involves additional cognitive skills, such as executive functioning (e.g., switching between tasks) and metacognition (e.g., recognizing similar tasks and solution patterns; Salomon and Perkins, 1987; Popat and Starkey, 2019). It is therefore key to further investigate the conditions and mechanisms underlying the possible transfer of the skills students acquire and the knowledge they gain during coding instruction via carefully designed learning interventions and experimental studies are needed that include the teaching, mediating, and assessment of transfer.

## Challenges With Measuring Cognitive Benefits

Despite the promising evidence on the cognitive benefits of learning to code, the existing body of research still needs to address several challenges to detect and document transfer effects—these challenges include but are not limited to Tondeur et al. (2019):

- Measuring coding skills. To identify the effects of learning-to-code interventions on coding skills, reliable and valid measures of these skills (e.g., performance scores) must be included. These measures allow researchers to establish baseline effects, that is, the effects on the skills trained during the intervention (Melby-Lervåg et al., 2016). However, the domain of computer coding largely lacks measures showing sufficient quality (Tang et al., 2020).
- Measuring other cognitive skills. Next to the measures of coding skills, measures of other cognitive skills must be administered to trace whether coding interventions are beneficial for learning skills outside the coding domain and ultimately document transfer effects. This design allows researchers to examine both near and far transfer effects and to test whether gains in cognitive skills may be caused by gains in coding skills (Melby-Lervåg et al., 2016).
- Implementing experimental research designs. To detect and interpret intervention effects over time, pre- and post-test measures of coding and other cognitive skills are taken, the assignment to the experimental group(s) is random, and students in the control group(s) do not receive the coding intervention. Existing meta-analyses examining the near and far transfer effects of coding have shown that these designs features play a pivotal, moderating role, and the effects tend to be lower for randomized experimental studies with active control groups (e.g., Liao, 2000; Scherer et al., 2019, 2020). Scholars in the field of transfer in education have emphasized the need for taking into account more aspects related to transfer than only changes in scores between the pre- and post-tests. These aspects include, for instance, continuous observations and tests of possible transfer over larger periods of time and qualitative measures of knowledge application that could make visible students' ability to learn new things and to solve (new) problems in different types of situations (Bransford and Schwartz, 1999; Lobato, 2006).

Ideally, research studies address all of these challenges; however, in reality, researchers must examine the consequences of the departures from a well-structured experimental design and evaluate the validity of the resultant transfer effects.

## Discussion

Overall, the evidence supporting the cognitive benefits of learning to code is promising. In the first part of this opinion paper, we argued that coding skills and other skills, such as creative thinking and mathematical problem solving, share skillsets and that these common elements form the ground for expecting some degree of transfer from learning to code into other cognitive domains (e.g., Shute et al., 2017; Popat and Starkey, 2019). In fact, the existing meta-analyses supported the possible existence of this transfer for the two domains. This reasoning assumes that students engage in activities during coding through which they acquire a set of skills that could be transferred to other contexts and domains (e.g., Lye and Koh, 2014; Scherer et al., 2019). The specific mechanisms and beneficial factors of this transfer, however, still need to be clarified.

The evidence we have presented in this paper suggests that students' performance on tasks in several domains other than coding is not enhanced to the same extent—that is, acquiring some cognitive skills other than coding is more likely than acquiring others. We argue that the overlap of skillsets between coding and skills in other domains may differ across domains and the extent to which transfer seems likely may depend on the degree of this overlap (i.e., the common elements), next to other key aspects, such as task designs, instruction, and practice. Despite the evidence that cognitive skills may be prompted, the direct transfer of what is learned through coding is complex and does not happen automatically. To shed further light on the possible causes of why transferring coding skills to situations in which students are required to, for instance, think creatively may be more likely than transferring coding skills to situations in which students are required to comprehend written text as part of literacy, researchers are encouraged to continue testing these effects with carefully designed intervention studies and valid measures of coding and other cognitive skills. The transfer effects, although large enough to be significant, establish some evidence on the relation between learning to code and gains in other cognitive skills; however, for some skills, they are too modest to settle on the ongoing debate whether transfer effects were only due to the learning of coding or exist at all. More insights into the successful transfer are needed to inform educational practice and policy-making about the opportunities to leverage the potential that lies within the teaching of coding.

## Author Contributions

RS conceived the idea of the paper and drafted the manuscript. FS and BS-S drafted additional parts of the manuscript and performed revisions. All authors contributed to the article and approved the submitted version.

## Conflict of Interest

The authors declare that the research was conducted in the absence of any commercial or financial relationships that could be construed as a potential conflict of interest.

## Publisher's Note

All claims expressed in this article are solely those of the authors and do not necessarily represent those of their affiliated organizations, or those of the publisher, the editors and the reviewers. Any product that may be evaluated in this article, or claim that may be made by its manufacturer, is not guaranteed or endorsed by the publisher.
